# The fingernail clams (Bivalvia: Veneroida: Sphaeriidae) of Morocco: Diversity, distribution and conservation status

**DOI:** 10.3897/BDJ.9.e73346

**Published:** 2021-12-02

**Authors:** Hanane Rassam, Mohamed Ghamizi, Hassan Benaissa, Catharina Clewing, Christian Albrecht

**Affiliations:** 1 Laboratory of Water, Biodiversity and Climate change, Faculty of Sciences Semlalia, Cadi Ayyad University, Marrakech, Morocco Laboratory of Water, Biodiversity and Climate change, Faculty of Sciences Semlalia, Cadi Ayyad University Marrakech Morocco; 2 Laboratory of Water, Biodiversity and Climate change, Faculty of Sciences Semlalia, Cadi Ayyad University, Marrakech. Fishing department, Marine Fisheries Technology Institute, Al Hoceima, Morocco Laboratory of Water, Biodiversity and Climate change, Faculty of Sciences Semlalia, Cadi Ayyad University, Marrakech. Fishing department, Marine Fisheries Technology Institute Al Hoceima Morocco; 3 Department of Animal Ecology & Systematics, Justus Liebig University Giessen, Giessen, Germany Department of Animal Ecology & Systematics, Justus Liebig University Giessen Giessen Germany

**Keywords:** *Pisidium*, *Musculium*, Moroccan basins, mountain biodiversity, morphometry, conservation, freshwater ecosystems

## Abstract

**Background:**

In Morocco, many uncertainties surround the current diversity and distribution of the bivalve family Sphaeriidae. Such information, including taxonomy and conservation status, are vital for further studies to improve the knowledge of this family in Morocco and represents the first step towards the development of a national conservation plan for all freshwater bivalves.

Between 2016 and 2019, several investigations were carried out to assess the diversity and distribution of Sphaeriidae in the different basins of Morocco, covering different types of habitat (lakes, springs, rivers and small ponds). The identification of specimens and their morpho-ecological features was based on morphological and morphometric analyses. The data on the general distribution of the collected species allowed the evaluation of their conservation status as well.

The shell morphology and morphometric analyses revealed the existence of five species belonging to the genus *Pisidium* [*P.casertanum* (Poli, 1791), *P.* (cf.) *personatum* Malm, 1855, *P.subtruncatum* Malm, 1855, *P.amnicum* (O. F. Müller, 1774) and *Pisidium* sp.] and one species to the genus of *Musculium* [*M.lacustre* (O. F. Müller, 1774)]. Sphaeriidae were found in all Moroccan basins, except Bouregreg and Sakia El Hamra-Oued Eddahab Basins. The results showed that Sebou Basin was the species richest with the occurrence of the five species, while Loukkos and Sous-Massa Basins had the lowest-species richness with one species only. The conservation status of sphaeriids in Morocco was evaluated for the first time and resulted in *P.* (cf.) *personatum* and *P.subtruncatum* being proposed as Least Concern and Vulnerable, respectively, while the status of Regionally Extinct is suggested for both species *P.amnicum* and *M.lacustre*.

**New information:**

First evaluation of the diversity and species richness of the family Sphaeriidae in Morocco with an assignment of an updated conservation status of the recorded species.

## Introduction

The North African region is globally identified as containing ecosystems with an important biodiversity (Cormier-Salem et al. 2018). Amongst them, freshwater ecosystems are reported to be particularly fragile and several native species are threatened and at risk of extinction due to habitat loss and degradation induced by anthropogenic pressure, pollution and climate change (Carpenter et al. 2011). Morocco possesses the most extensive river system in North Africa (Di Piazza 2006). The precipitation that falls in the high mountain ranges of the Rif, Middle Atlas, high Atlas and Anti-Atlas feeds rivers generally flowing north-westwards to the Atlantic or south-eastwards towards the Sahara. Moulouya River is the main exception, flowing from the Middle Atlas to the Mediterranean Sea (Barrios et al. 2014).

One of the freshwater taxa present in these rivers is the family Sphaeriidae, commonly referred to as fingernail and pea clams, including the tiniest freshwater bivalves in the world with lengths from 2 to 25 mm (Frankiewicz 2018) and a worldwide distribution, except for Antarctica (Thorp and Covich 2010, Williams et al. 2014). Currently, the family counts more than 220 species that often dominate benthic communities in aquatic ecosystems including rivers, streams, lakes and even ephemeral pools (Herrington 1962, Kuiper 1983, Sousa et al. 2011, Burch 1975). They can be of high importance in the ecosystem such as their significance to the phosphorus cycle (Kasprzak 1986). Species of the family Sphaeriidae can be of great interest in assessing and monitoring the environmental conditions of a given ecosystem and some species are useful as bioindicators of the trophic status of lakes (Clarke 1979). They are a major component of the diet of many waterbirds and bottom-feeding fish (Baker 1982, Brown 2009, Gale 1973). Sphaeriidae, therefore, enhance the survival and functioning of freshwater ecosystems. Fossils of Sphaeriidae are important in paleontological studies by enabling the reconstruction of ancient habitats (Herrington 1962). The fingernail clams are known to have a high passive dispersal capacity by attaching to other animals (insects, fish, amphibians and mammals) (Clewing et al. 2013, Mackie 1979). The high intra-specific shell variability and the subtlety of differences between species can lead to misidentification of species (Kilgour et al. 1990, Rassam et al. 2021b). The nomenclature within the Sphaeriidae family has been discussed many times and genera included in the Sphaeriidae family are not yet definitive and different studies have recovered *Musculium* as a subgenus nested within *Sphaerium* (Bogan 2013, Graf and Cummings 2021). However, the subclade assemblages of *Pisidium* (*Afropisidium* Kuiper, 1962, *Odhneripisidium* Kuiper, 1962, *Euglesa* Jenyns, 1832 and *Pisidium* s. str.) were based on molecular genetic data recovered as subclades (Clewing et al. 2013, Lee 2019, Lee and Foighil 2003, Schultheiß et al. 2008). To date, the taxonomic status of these subclades is still controversial (see Lee 2019) and a thorough taxonomic revision of the genus *Pisidium* is necessary.

The North African Region represents a biogeographic transition between the Palearctic and Afrotropical realms. The molluscan biogeography of the North African Region is well-defined. On the basis of the composition of mollusc communities since the beginning of the Holocene, Damme (1984) divided North Africa into two parts belonging to the Palearctic and Afrotropical (= Ethiopian) Regions, respectively. Therefore, the study of the malacological composition is of great interest to increase the knowledge on that region. In comparison to the Moroccan unionids (Araujo et al. 2009a, Araujo et al. 2009b, Benaissa et al. 2019, Froufe et al. 2016a, Froufe et al. 2016b, Gomes-dos-Santos et al. 2019, Sousa et al. 2016, Sousa et al. 2017), sphaeriids have received less attention and mentions of this family were limited to the faunistic studies with other groups of aquatic invertebrates (Talami 1998) and records of occurrence in some areas in Morocco (Kuiper 1972, Rassam et al. 2020). The first works on malacology in Morocco referred to species of *Pisidium* under the following names: *P.atlasicum* (Pallary, 1915), *P.rotundatum* (Pallary, 1921), *P.marocanum* (Pallary, 1936), which are considered synonymous with *P.casertanum* (Poli, 1791) (Kuiper 1964) and *P.marteli* (Pallary, 1927) which is a synonym of *P.amnicum* (Müller, 1774) (Kuiper 1966a). As a result, only *P.casertanum* and *P.amnicum* were known in Morocco before Kuiper (1972), who added five other species to the list: *P.milium* Held, 1836, *P.personatum* Malm, 1855, *P.subtruncatum* Malm, 1855, *P.nitidum* (Jenyns, 1832) and *P.tenuilineatum* Stelfox, 1918. However, key information for the Moroccan sphaeriid fauna, including taxonomy, diversity and distribution, are still lacking. Furthermore, the conservation status of only large mussels in Morocco has been comprehensively assessed over the past decades, with the most recent assessment published in 2019 (Gomes-dos-Santos et al. 2019). However, only large mussels have been studied. Consequently, the conservation status of the family Sphaeriidae has not been assessed (since the 2010 assessment by the IUCN Red List (Damme et al. 2010). The International Union for the Conservation of Nature (IUCN) Red List criteria are “The global standard for assessing extinction risk” (IUCN 2012). One of the most widely used IUCN Red List assessment criteria is the geographical extent (Criterion B) which is a strong predictor for extinction risk measured by the extent of occurrence (EOO, Criterion B1) and area of occupancy (AOO, Criterion B2) (Gaston and Fuller 2009).

Given the importance and the lack of information on the basic bio-ecological features of Sphaeriidae in Morocco, this study aims to: i) present a first evaluation of the diversity of Sphaeriidae in Morocco, including species richness and composition, using morphometric and geometric analysis, ii) assess the distribution pattern of Sphaeriidae in Moroccan freshwater basins and iii) assign a conservation status to the Moroccan species of Sphaeriidae.

## Materials and methods

### Study area and field sampling

**Figure 1.**
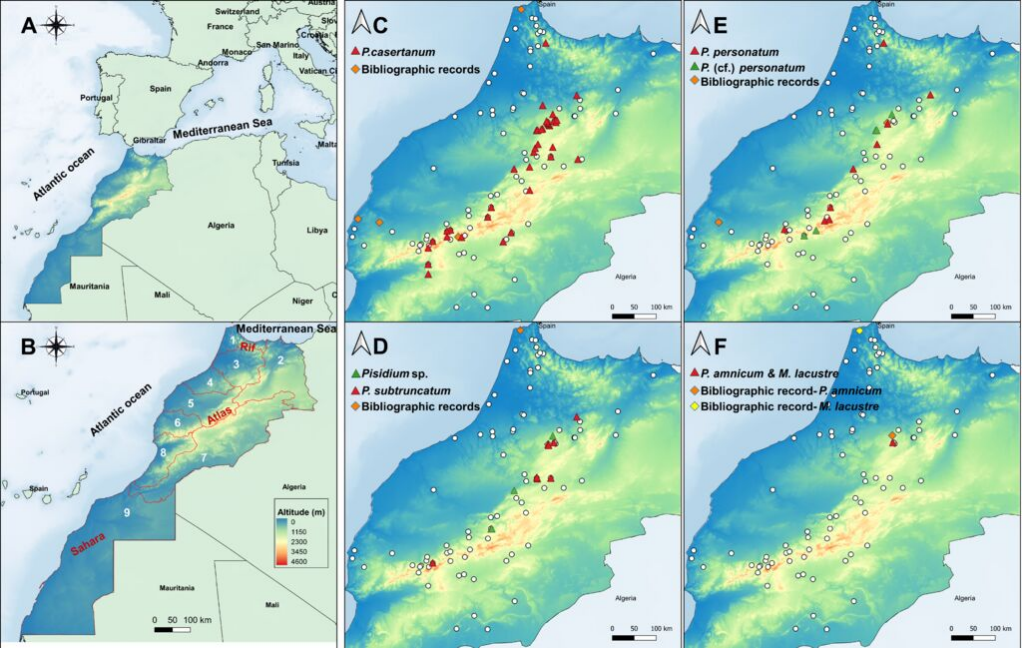
Location of the study area with sampling sites. **A** Geographic position of Morocco. **B** Map of Morocco with different basins assigned with numbers following the national division of the basins; 1: Loukkos Basin, 2: Moulouya Basin; 3: Sebou Basin, 4: Bouregreg Basin, 5: Oum Er Rabia Basin, 6: Tensift Basin, 7: Drâa-Ziz-Rhériss Basin, 8: Souss-Massa Basin, 9: Sakia El Hamra- Oued Eddahab. **C -F** Distribution maps of all five species (*M.lacustre* and *P.amnicum* occur in the same locality) across the country with their respective bibliographic records (marked with rhombi) (coordinates are given in Suppl. material 1), white circles represent the sampling sites with no specimens found.

Between 2016 and 2019, a total of 164 sampling sites were investigated covering the nine hydrological basins of Morocco (Fig. 1). The study area is characterised by mountain chains consisting of the Rif Mountains in the north, the Atlas in the middle part and the Sahara in the south of Morocco. The climate is very diverse; the northern coastal region part is characterised by a Mediterranean climate, with precipitation reaching 800 mm per year (El Moçayd et al. 2020), while in the southern part, a semi-arid and desert climate predominates with precipitation barely reaching 100 mm per year (El Moçayd et al. 2020).

Extensive sampling of sphaeriids covered all types of freshwater habitats (river systems, streams, lakes, reservoirs, ponds and marshes) using a sieve of 200 µm pore diameter. The sampling was conducted to the possible extent in reachable areas with the highest probability of occurrence. Localities mentioned in bibliographic records of the presence of some species of Sphaeriidae were also visited and checked according to Kuiper (1964), Kuiper (1972), Pallary (1898), Pallary (1915), Pallary (1921), Pallary (1927) and Pallary (1936) (Fig. 1). All specimens were immediately stored in 80% ethanol.

Species were identified on the basis of the main morphological characters (e.g. hinge plate, shell shape, ligament pit) using, for example, the identification keys of Sphaeriidae presented by Adam (1960), Piechocki (1989), Killeen et al. (2004), Glöer (2020) and Korniushin and Hackenberg (2000).

### Morphometric analysis and geometric morphometrics

For morphometric analysis, the specimens of different habitats and localities were photographed using a digital microscope system (Keyence VHX-2000). Seven variables were selected and measured on the three shell axes using the tpsDig v.2.17 programme (Rohlf 2017): LA (anterior length), LP (posterior length), L (total shell length), LL (ligament length), LH (hinge length), LE (length of umbo), HH (hinge height) and H (total shell height). The measurements followed the methodology used in Rassam et al. (2021b).

From these measurements extracted from the photographs, a series of ratios was derived: hinge length/shell length (**LH/L**), shell width/shell length (**W/L**), shell height/shell length (**H/L**), hinge height/shell height (**HH/H**), ligament length/shell length (**LL/L**) and anterior length/posterior length (**LA/LP**). The statistical package PAST v.4.06 (Hammer et al. 2001) was used to test differences between taxa, based on the measured ratios.

For outline analysis, 60 semi-landmarks were marked on the 30 right valves in order to draw a 2-dimensional mean shape for each of the three species using tpsrelw v.1.70 (Rohlf 2019). Note that the measurements of *P.amnicum* and *M.lacustre* were not included in the PCA analysis as the number of individuals of both species was less than the required minimum.

### Diversity data processing

To identify the spatial distribution of Sphaeriidae in the Moroccan basins, the georeferenced records were projected on the map using QGIS software v. 3.4.1 (2018). “Between basins” comparison of the species composition was implemented using Jaccard’s Similarity Index, working with presence-absence data using the following expression:

C_j_= a/(a+b+c),

where a is the number of species shared between the two compared sites, b and c are the number of species exclusive to site 1 and site 2, respectively. The index value goes from 0 (no similarity) to 1 (identical). A dendrogram was plotted using PAST v.4.06 (Hammer et al. 2001), showing the relationship between the different basins, based on the similarity of species.

### Conservation status assessment

The geographic range (criteria B) was used to evaluate the IUCN Red List category of Sphaeriidae in Morocco, based on the regional IUCN Red List guidelines. The extent of occurrence (EOO) and area of occupancy (AOO), respectively were defined according to the IUCN Red List (IUCN 2019) as "the area contained within the shortest continuous imaginary boundary which can be drawn to encompass all the known, inferred or projected sites of present occurrence of a taxon, excluding cases of vagrancy", whereas AOO is "the area within its 'extent of occurrence', which is occupied by a taxon, excluding cases of vagrancy". EOO and AOO were calculated for all the species, based on the occurrence points, using ConR package on R (Dauby et al. 2017), with a 2 km grid as the IUCN Red List default setting. AOO was obtained on a basis of 100 m buffer along the sampling sites. The values of AOO and EOO were then used, in addition to the number of locations and the continuing decline and/or the extreme fluctuations in the sub-criteria, automatically generated by the programme on R (Dauby et al. 2017), to assess the conservation status of each sphaeriid species in Morocco. For this analysis, only species identified were considered; *Pisidium* sp. was not included in the assessment (it is not yet clear to which species the specimens belong).

## Checklists

### *Pisidium* Pfeiffer, 1821

#### Pisidium casertanum

(Poli, 1791)

93D7D7AA-9D4A-5C37-B890-7589A20E1624

##### Ecological interactions

###### Native status

Autochtonous

##### Distribution

Cosmopolitan. More widely present in the Northern Hemisphere than the Southern one where it is more limited to high altitudes localities (Kuiper 1983). Widespread throughout Morocco.

##### Notes

Shell dimensions: Mean length = 4.09 ± 0.7 mm; Mean height = 3.42 ± 0.59 mm; Mean width = 2.52 ± 0.76 mm.

Key features: Extremely variable, but can be separated from other species by its flattened umbo and shell (Fig. 6). Periostracum silky, coated with ferruginous deposits. The dorsal margin is relatively long. Cardinal teeth: C2 arched, C4 straight and short, C3 strongly curved, at times bifurcated posteriorly.

Remarks: The species is known to be highly variable morphologically (Rassam et al. 2021b). The different morphological forms that the species can take are related to the effect of habitat conditions (Piechocki 1989).

#### Pisidium subtruncatum

Malm, 1855

A79549B4-EFDB-54F1-9F95-4E41506806C9

##### Ecological interactions

###### Native status

Autochtonous

##### Distribution

Holarctic. Found in Europe, North Africa, Siberia to Baikal Lake and North America (Ellis 1978).

##### Notes

Shell dimensions: Mean length = 2.92 ± 1.23 mm; Mean height = 2.45 ± 1.08 mm; Mean width = 1.74 ± 1.37 mm.

Key features: Subtriangular shape with a striated shell, opisthogyrous umbo (Fig. 7). Posterior end truncated and anterior part elongated. The hinge plate is relatively thick and arched. The left valve has 2 long parallel cardinal teeth (C2, C4). C3 is relatively straight and slightly curved, while lateral teeth are well-developed.

#### Pisidium personatum

Malm, 1855

03D8325B-A730-5B18-8BE0-6089181B3E30

##### Ecological interactions

###### Native status

Autochtonous

##### Distribution

Europe, Asia and North Africa (Kuiper 1983)

##### Notes

Shell dimensions: Mean length = 2.92 ± 0.7 mm; Mean height = 2.45 ± 0.6 mm; Mean width = 1.74 ± 0.88 mm.

Key features: Rounded shape, centrally located umbo, but not prominent. The presence of a raised callus in the hinge is a specific feature of the species (Fig. 8), lying between the lateral teeth and the ligament pit. The callus is mainly present in the right valve and may be absent in the left valve.

Remarks: *Pisidiumpersonatum* may be confused with *P.casertanum* as both species share similar morphological features, but *P.personatum* can be easily identified from other species of *Pisidium* by the presence of a callus in the hinge plate between the ligament pit and the lateral teeth on both valves, although it is less marked on the left valve.

#### Pisidium amnicum

(O. F. Müller, 1774)

A6A6F029-80D9-53EC-8E8F-CBF423C0DB65

##### Ecological interactions

###### Native status

Autochtonous

##### Distribution

Palearctic. More common in the north of Europe than in the south. In Morocco, it was reported in the present study only from a reservoir outlet.

##### Notes

Shell dimensions: Mean length = 8.5 ± 1.5 mm; Mean height = 6.96 ± 1.24 mm.

Key features: Large size (up to 10 mm) and clearly marked striations on the shell, irregularly spaced and denser near the umbo. The umbo is not prominent, placed posteriorly (Fig. 9b). The hinge plate is thick. C2 triangular with shorter C4, C3 is triangular and often bifurcated.

Remarks: *Pisidiumamnicum* remains the largest representative of *Pisidium* and, thus, it is always easy to distinguish.

#### Pisidium sp.

C9ADD64B-BC78-587C-B053-D9BC682805B3

##### Notes

Overall, no external distinguishing morphological features. However, the shell outline and hinge plate are closer to *P.casertanum* (Fig. 10).

### *Musculium* Link, 1807

#### Musculium lacustre

(O. F. Müller, 1774)

288E1846-ACD3-5C31-84FE-1469475F71EA

##### Ecological interactions

###### Native status

Autochtonous

##### Distribution

Holarctic

##### Notes

Shell dimensions: Length = 5.91-5.41 mm; Height = 4.71-4.45 mm

Key features: Quadrangular shell formed with a cap-like umbo centrally placed (Fig. 9a). The shell is very thin, fragile and smooth. The hinge plate is very narrow and cardinals very small.

## Analysis

### Morphometry

**Figure 2.**
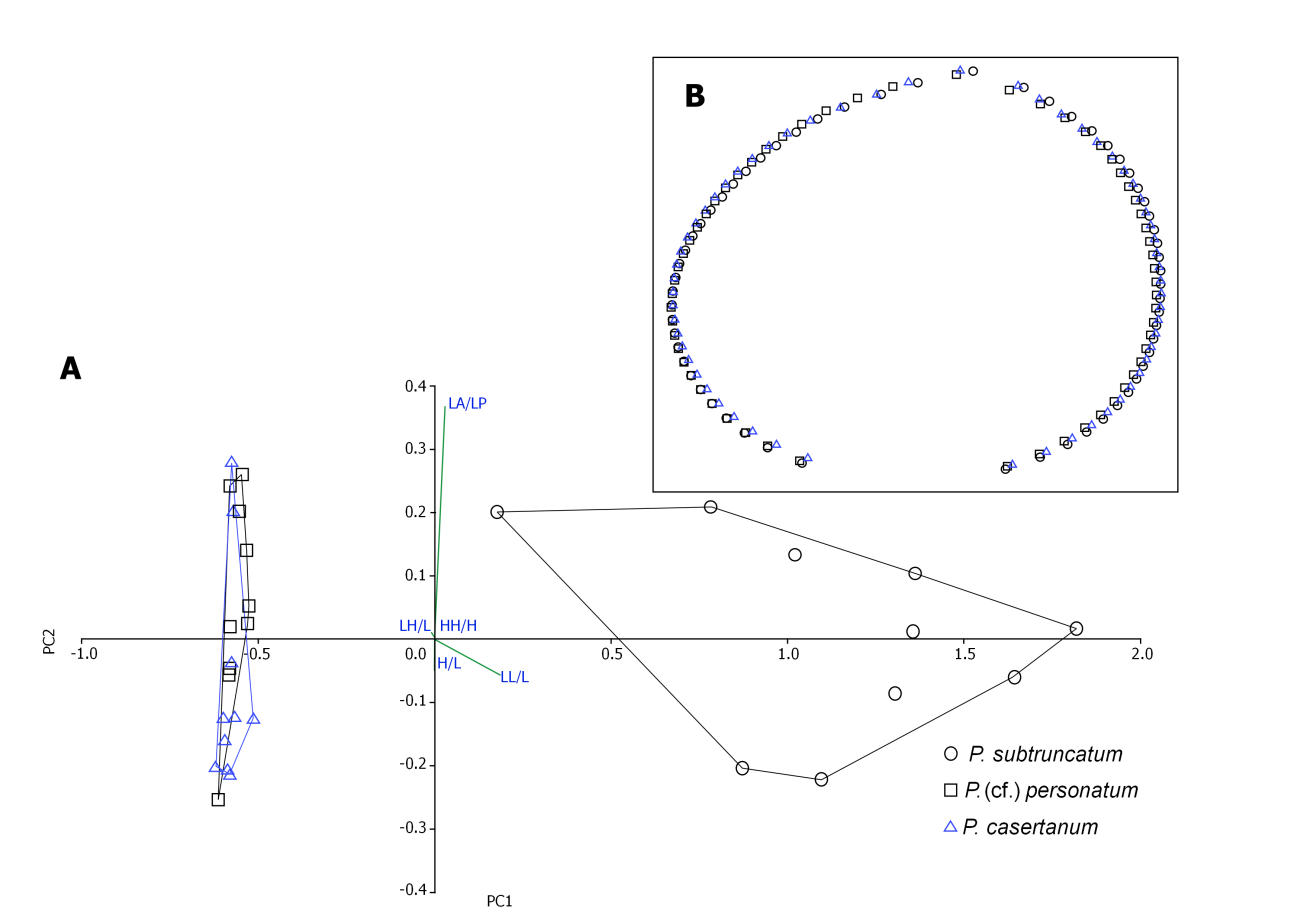
Results of the morphometric analysis of three species of Sphaeriidae from Morocco. **A** Principal Component Analysis (PCA) with variables that contributed most to the PCA analysis, based on the measured ratios. Length L (maximum distance on the anterior – posterior axis), length of anterior part LA, length of posterior part LP, height H (maximum distance on the dorsal-ventral axis), length of umbo LU, length of the ligament of the left valve LL, length of the hinge LH, height of the hinge of the left valve HH; **B** Mean outline of semi-landmarks of the three species (species symbols as in A).

The morphological diagnosis and morphometric analysis of the sampled specimens were of great usefulness as they permitted the identification and discrimination of species, based on the different morphometric ratios calculated as shown in Fig. 2. The PCA result showed two distinct groups where the first included *P.subtruncatum* and the second included both *P.personatum* and *P.casertanum*. A clear difference was revealed between *P.subtruncatum* and the other two species. The most discriminant ratios were LL/L and LA/LP (defining, respectively, the size of the ligament pit and the global shape of the shell), while *P.casertanum* and *P.* (cf.) *personatum* are somewhat confounded (Fig. 2-A). The 61 traced semi-landmarks enabled an overall outline of the shells of the three species to be generated. The differences between the mean outline of the three species are shown in Fig. 2-B. The greatest differences observed were in the areas of the umbo and the anterior ventral margin, i.e. *P.personatum* has a rounded shape and a flattened umbo, while *P.subtruncatum* has a posteriorly-orientated umbo and a relatively elongated anterior ventral margin.

### Species diversity and distribution

**Figure 3.**
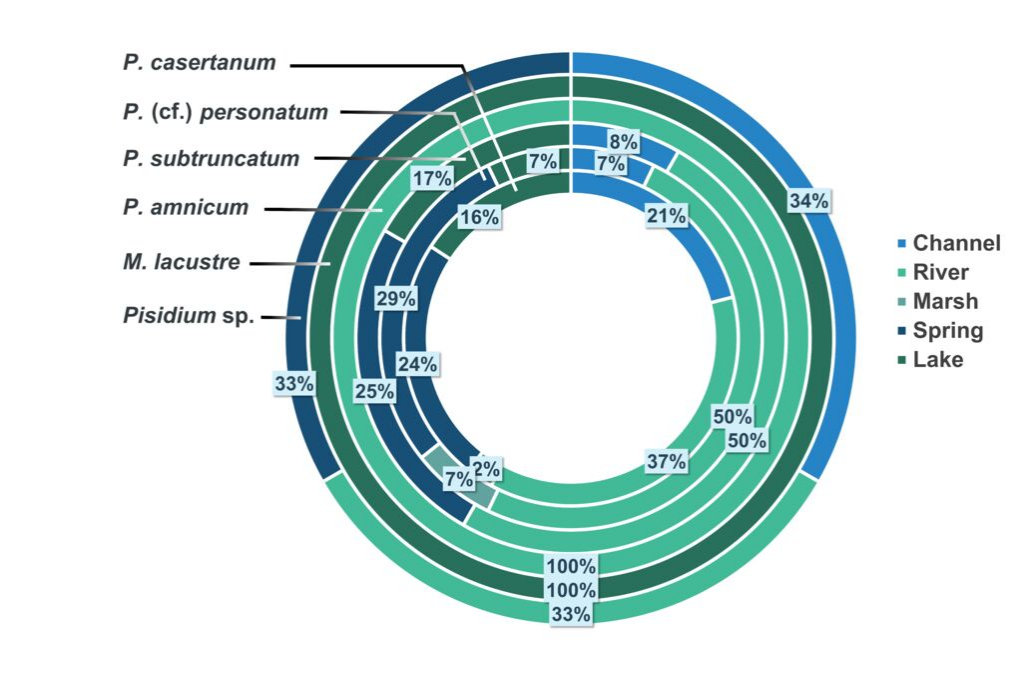
Frequency of species of Sphaeriidae by habitat type (n = 56 localities).

More than 164 sites were sampled during the working period, out of which 56 were inhabited by species of Sphaeriidae (see Suppl. material 1). A total of six species of Sphaeriidae were found in the freshwater habitats of Morocco, represented by two genera: *Pisidium* (*P.casertanum*, *P.* (cf.) *personatum*, *P.subtruncatum*, *P.amnicum* and *Pisidium* sp.) and *Musculium (M.lacustre)* (*Suppl. material 1*). *Pisidium* (cf.) *personatum* refers to specimens that are very close to *P.personatum*, but need confirmation. They both share the same morphological characters; however, they formed separate although closely-related genetic clades. *Pisidium* sp. refers to specimens that showed clear genetic differences to the other species and, thus, cannot be given a specific name. Genetic data (Rassam, Clewing, Albrecht, unpublished data) revealed that this clade differs from all other species described for Morocco which suggests that it may be a different and potentially new species. *Pisidiumcasertanum* was the most abundant species occurring in all the habitat types, followed by *P.* (cf.) *personatum* and *P.subtruncatum. Pisidiumamnicum* and *M.lacustre* ranked in last position occurring together in only one locality. Riverine habitats represented the ecosystems most frequently inhabited by Sphaeriidae (35% of the total habitats), followed by springs with 30%. Marshes were the least represented with only 2% (Fig. 3).

In terms of species richness, the Sebou Basin ranked first with the occurrence of all six species, whereas Souss-Massa and Moulouya had the lowest species number with only one species found. The Bouregreg and Sakia El Hamra-Oued Eddahab Basins were exceptional since no sphaeriids were found.

**Figure 4.**
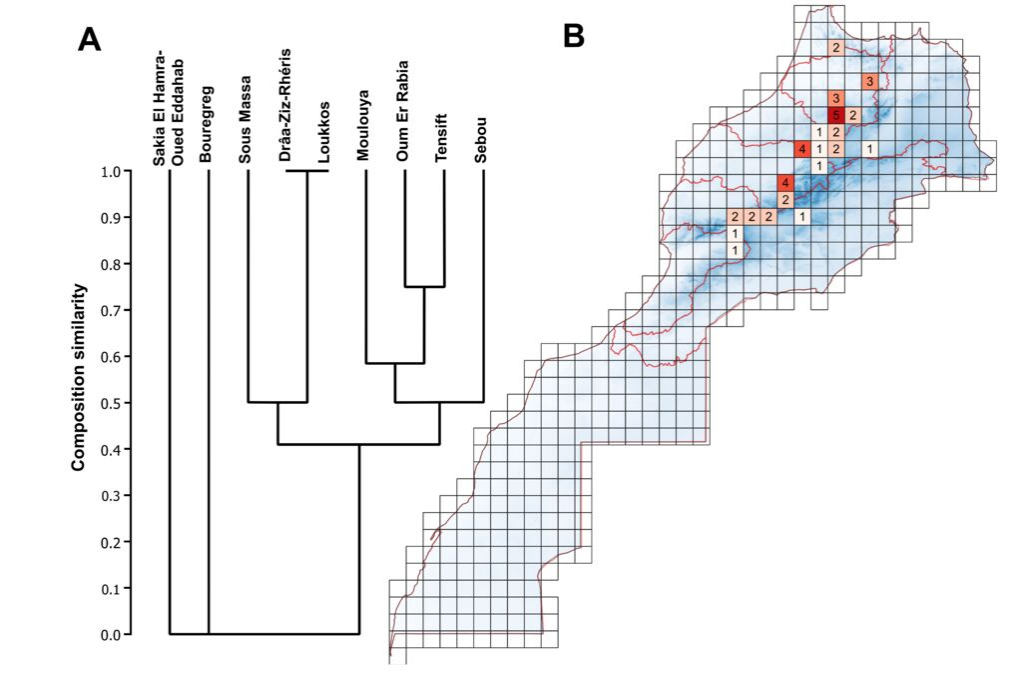
Species richness by basin. **A** Dendrogram of species composition by basin using Jaccard’s Similarity Index. **B** Grid map 50 x 50 km showing the geographical distribution of species by basin.

*Pisidiumcasertanum* was found in all the basins except Bouregreg and Sakia El Hamra-Oued Eddahab. *Pisidium* (cf.) *personatum* occurred in five basins: Loukkos, Tensift, Sebou, Souss-Massa and Oum Er Rabia. *Pisidiumsubtruncatum* was recorded in four basins: Tensift, Sebou, Moulouya and Oum Er Rabia. *Pisidium* sp. was present in both Oum Er Rabia and Sebou Basins, whereas *P.amnicum* and *M.lacustre* were only reported from Sebou Basin (Fig. 4).

### Conservation status

Extent of Occurrence and Area of Occupancy differed significantly amongst the regional populations of the species. EOO ranged from 15,219 km^2^ to 50,915 km^2^, while AOO ranged between 40 km^2^ and 104 km^2^. Table 1 gives the detailed results of the assessment with the assigned Red List categories to each species. *Pisidiumcasertanum* had the highest EOO and AOO across the country amongst the different species of Sphaeriidae, followed by *P.* (cf.) *personatum*, while *P.subtruncatum* was the least commonly present amongst the five assessed species and is, therefore, the most vulnerable one (Table 1). *Pisidiumamnicum* and *M.lacustre* were listed as critically endangered species. However, only empty shells of these two species were included in the analysis; thus, the results for their conservation status cannot be determined accurately.

## Discussion

### Species diversity

The results of the present study confirmed the existence of at least six sphaeriid species occurring in different basins in Morocco. These findings have cleared up doubts about the existence of other species mentioned in literature as synonyms or living species in Morocco.

All the seven species recorded by Kuiper (1972) co-existed in a single locality in the Middle Atlas (Sebou Basin) and some specimens were deposited in the Natural History Museum of the Scientific Institute in Rabat (Rassam et al. 2021a); therefore, only four out of the seven species were found in the whole country. Given the overall comparatively low diversity of Sphaeriidae in Morocco, it is interesting that we discovered a new species for the fauna of the country. Moreover, it is interesting to see a species potentially new to science. New species of Sphaeriidae are rarely described in the Palaearctic (e.g. Groh et al. 2020). Detailed analyses must assess whether *Pisidium* sp. is indeed an endemic to Morocco.

The difference in the number of species mentioned for Morocco may have several possible explanations: the identification on which the authors relied at the time was based on morphological and anatomical features; however, the genus *Pisidium* is cryptic and its species show pronounced intra-specific variations, influenced by environmental conditions (Funk and Reckendorfer 2008, Holopainen and Hanski 1986, Holopainen and Kuiper 1982, Rassam et al. 2021b), thus, the identification may be confounded in the absence of clear morphological features. These can be seen, however, with the combination of both morphological and geo-morphometric approaches. The species *P.tenuilineatum*, for example, is easily confused with *P.subtruncatum*, based on the outline shape of the shell (Piechocki 1989) and has never been reported from altitudes above 500 m a.s.l. (while Kuiper's record of the species is above 1,700 m), suggesting that this is potentially a misidentification. The second possible explanation is the change in environmental conditions; a study by Økland and Kuiper (1982) showed the impact that water acidification can have on the disappearance of *Pisidium* species. *Musculiumlacustre* was cited for the first time in Morocco as *Sphaeriummaroccanum* Pallary, 1898 in the surroundings of Tangier, a city in the north-west; thus, it was thought to be restricted in Africa to the Algerian coast (Damme 1984).

### Distribution patterns and conservation status

Sphaeriids were collected in different habitat types, including lakes and reservoirs, springs, channels, marshes and rivers (streams, dam outlets and larger tributaries). River systems were the habitat with the greatest abundance of Sphaeriidae.

*Pisidiumcasertanum* was the most abundant species of sphaeriid in all habitat types. This species is euryeceous and is, therefore, considered the most common member of the family. It can be found in almost all habitat types ranging from temporal and ephemeral ponds to large rivers and lake bottoms (Clarke 1973, Saunders and Rung 1990). This species is also known to be tolerant in terms of environmental conditions, such as low pH values and low calcium concentrations (Horsák and Hájek 2003, Økland and Kuiper 1982); consequently, its occurrence in all habitat types in this study is not surprising. *Pisidiumpersonatum* is present in all habitats sampled, but is more concentrated in springs and rivers. *Pisidiumpersonatum* is a typical cold-stenoecous inhabitant of springs and wells (Dyduch-Falniowska 1982, Wagner et al. 2011) and seeks nutrient-rich sites (Horsák et al. 2007, Kubíková et al. 2011), which explains its abundance in marshy areas (Fig. 2). *Pisidiumamnicum* occurred in only one locality which was a small outlet of a dam reservoir in the Sebou Basin, where only a few empty shells were found. The species is not flexible in terms of water quality and prefers oligosaprobic and mesosaprobic waters (Piechocki 1989); moreover, it is abundant especially in northern and Central Europe (Zettler and Daunys 2007, Zettler 1996) and its presence in Morocco may be an introduction, but more research is necessary to test this hypothesis. These two characteristics may explain its rarity in the samples. *Pisidiumsubtruncatum* occurs in all types of habitats surveyed, except marshes, with a low abundance in rivers. It is an euryeceous species which ranks behind *P.casertanum* and *P.personatum* in the variety of its habitats. *Musculiumlacustre* is the only representative of its genus in Morocco, its representation is nevertheless not very pronounced, as only two old valves were found in a dam reservoir with submerged vegetation. The habitat record is in harmony with those of Swanson and Ormerod (2005) and Killeen et al. (2004); however, the very small number of individuals collected indicates thatresampling in this same locality should be undertaken more intensively.

The distribution of species of Sphaeriidae in Morocco is uneven across the nine basins and across the different altitudes. The highest diversity is recorded in the Middle Atlas which links the Sebou and Oum Er Rabia Basins with the presence of all the five species in the 50 x 50 km square around the city of Ifrane. The Middle Atlas is known for its richness in aquatic resources mainly from snowmelt and heavy rainfall, resulting in a variety of ecological habitats (springs, lakes, tributaries, marshes and large rivers), which may explain the specific diversity that occurs in this region.

**Figure 5.**
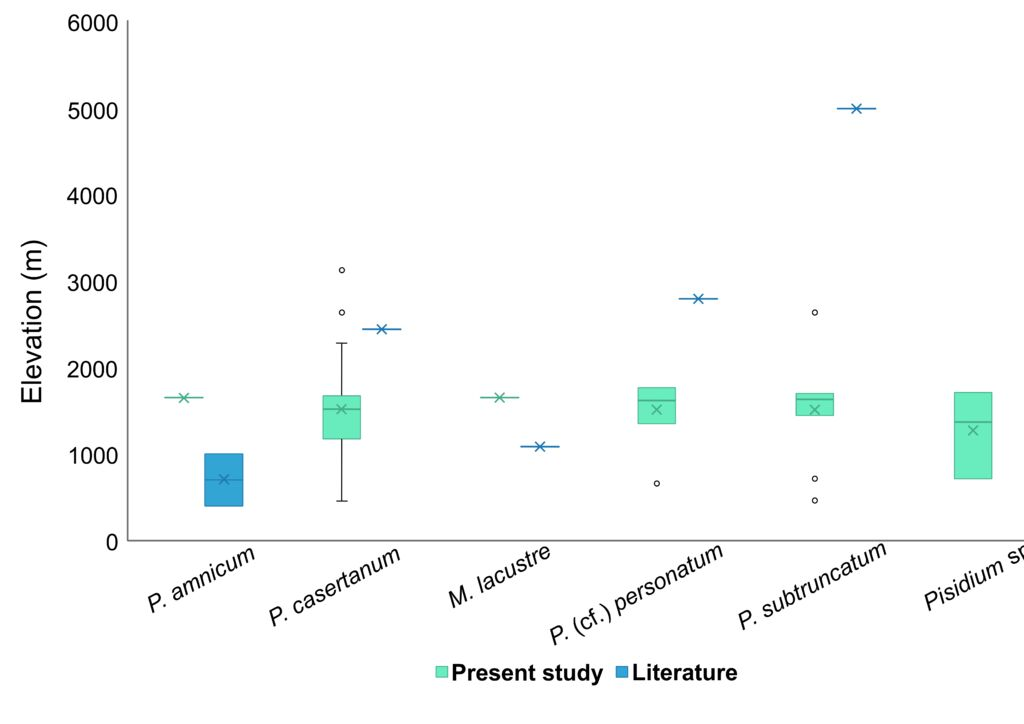
Altitudinal distribution range of species of Sphaeriidae: *P.amnicum* (Kuiper 1972, Hubenov 2007); *P.casertanum* (Mouthon 1983); *M.lacustre* (Piechocki 1989); *P.personatum* (Kuiper et al. 1989); *P.subtruncatum* (Clewing et al. 2013) (the crossmarks and the horizontal bars on the boxes refer to the mean and median, respectively).

**Figure 6.**
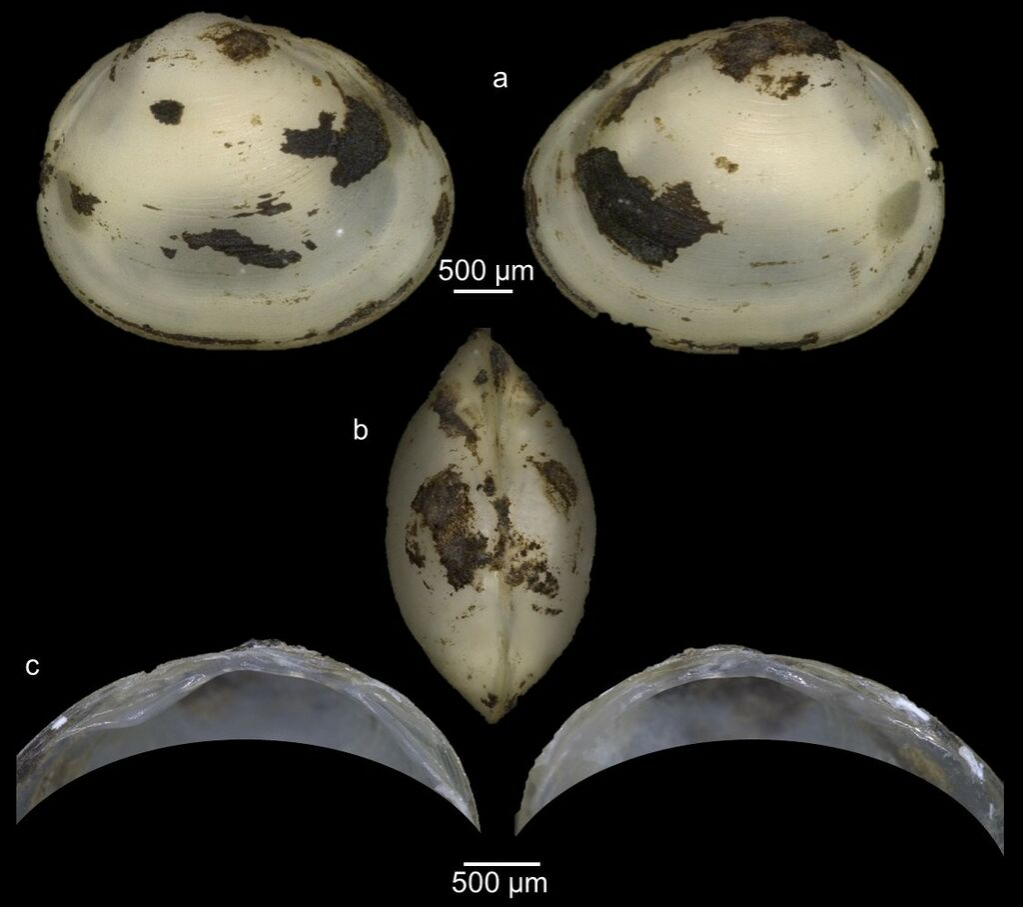
*Pisidiumcasertanum* from Ouzioua River **a** External view of the whole animal (left and right); **b** Dorsal view; **c** internal view of hinge plate of left and right valves.

**Figure 7.**
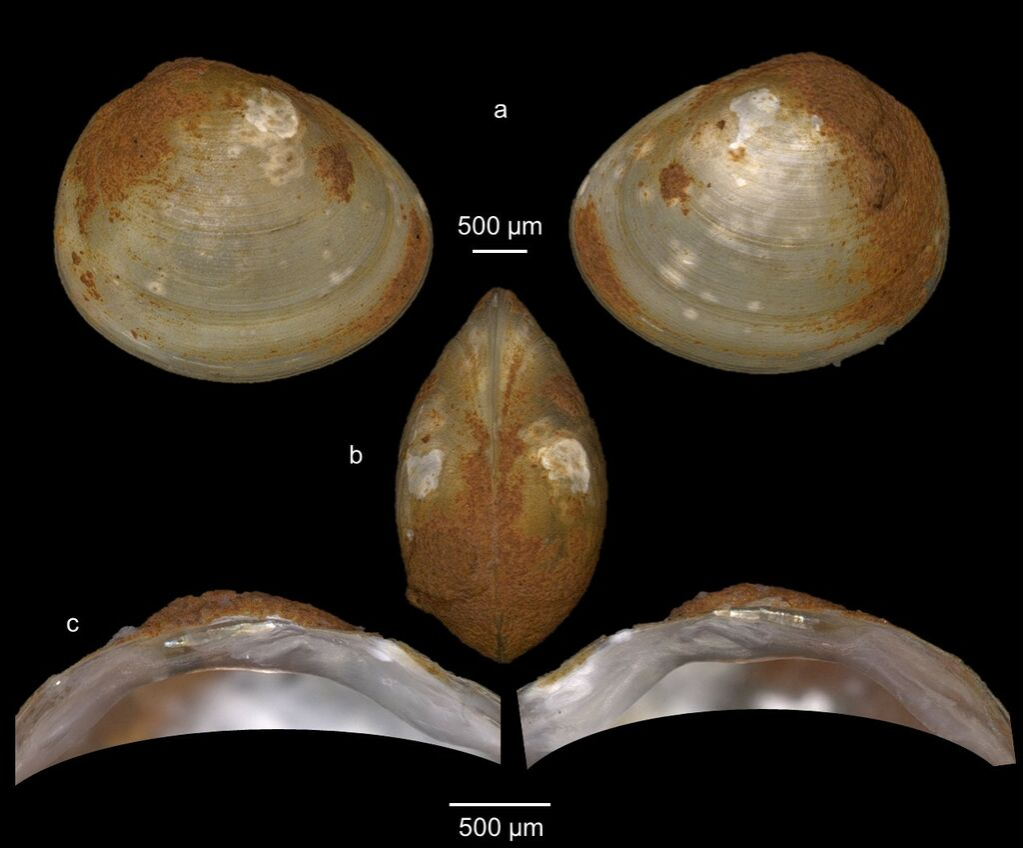
*Pisidiumsubtruncatum* from Fendel spring **a** External view of the whole animal (left and right); **b** Dorsal view; **c** internal view of hinge plate of left and right valves.

**Figure 8.**
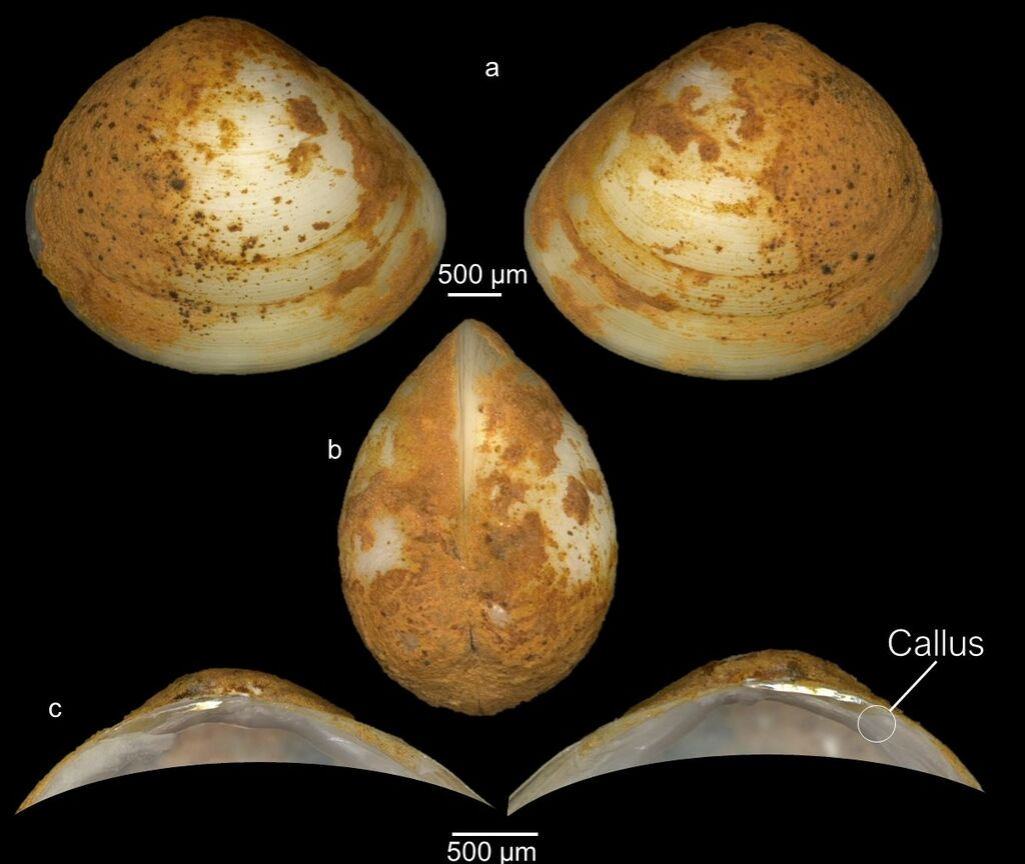
*Pisidiumpersonatum* from Tessaout River **a** External view of the whole animal (left and right); **b** Dorsal view; **c** internal view of hinge plate of left and right valves.

**Figure 10.**
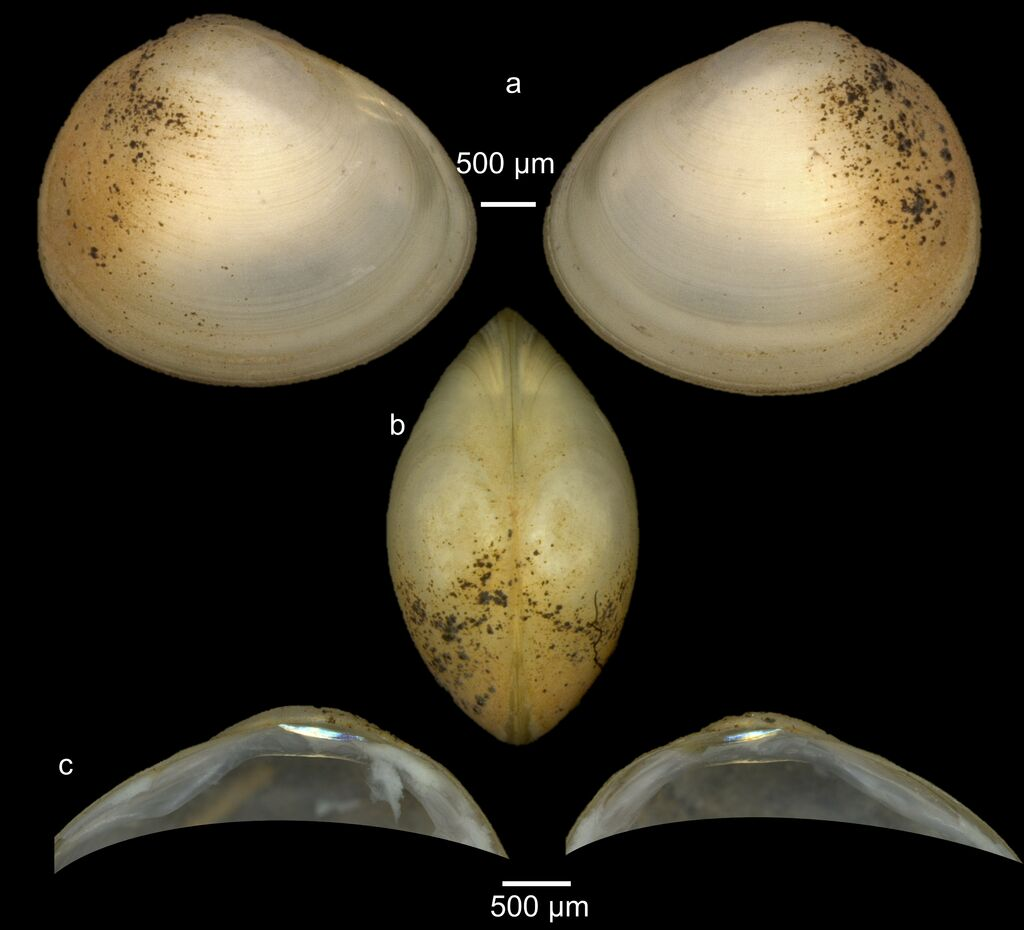
*Pisidium* sp. from Zaouit Cheikh channel. a- External view of the whole animal (left and right), b- Dorsal view, c- internal view of hinge plate of left and right valves.

With respect to elevation, the highest diversity of sphaeriids species in Morocco was found between 1,000 and 2,000 m a.s.l. in the Sebou Basin. No species were recorded below 462 m. *Pisidiumcasertanum* is the species that showed a wide altitudinal spectrum ranging from 462 to 3,137 m a.s.l. Despite being a lowland species (Piechocki 1989, Moorkens and Killeen 2009), *P.amnicum* was found in our study at a relatively high altitude (1663 m a.s.l.). In Europe, *P.subtruncatum* has been recorded at a maximum altitude of 2,300 m in the Pyrenees (Kuiper 1966b); in this work, this limit is exceeded to 2,645 m a.s.l. in the Tensift Basin. Globally, *P.subtruncatum* has the highest point of occurrence in the Tibetan Plateau (Clewing et al. 2013). *Pisidiumpersonatum* has a narrower altitudinal range (Fig. 5) with a maximum of 2,175 m a.s.l. which does not exceed that recorded by Kuiper (1974) in the Alps (2,500 m a.s.l.). The difference in distribution and species composition of Sphaeriidae between basins may be explained by the fact that the Sebou Basin, which is the richest in species, is one of the largest in Morocco (40,000 km^2^) and, therefore, greater diversity could be expected (Kallimanis et al. 2008). A second explanation for the altitudinal distribution may be the fact that the Sebou, Oum Er Rabia and Tensift Basins are the highest basins of the country, the presence of mountain chains crossing these regions providing a variety and diversity of habitats.

The conservation status following the IUCN Red List guidelines was elaborated for the first time at the national level for all the species of Sphaeriidae recorded in Morocco. Out of the five species, *P.casertanum* and *P.personatum* have been assessed as “Least Concern” species in Morocco. *Pisidiumpersonatum* was previously assessed by the IUCN Red List at the North African scale as Vulnerable (Damme et al. 2010). The conservation status assessment of *P.subtruncatum* classified the species as “Vulnerable”, while at the North African scale, it was assessed as “Critically Endangered” (Damme et al. 2010). The preliminarily-suggested status for *P.amnicum* and *M.lacustre* at the national level is "Regionally Extinct", while at the North African level, the species is in the "Data Deficient" class (Damme et al. 2010) as exhaustive sampling has failed to record any living animals; moreover, only empty shells of *M.lacustre* were collected in one locality which is nowadays completely dry. The conservation status remains challenging to assess without estimation of the species abundance in unsurveyed areas. Modelling, taking into account unsurveyed areas along with other factors, such as climate oscilliations and threats to species, remains essential to assess the conservation status of species. In the present work, the sampling covered, to the extent possible, the reachable sites corresponding to the habitat of the Sphaeriidae. The unsurveyed areas, although limited in number, were included in the analysis as a part of the whole study area and the results obtained represent a preliminary dataset to help set up a normalised regional conservation status. This is an important task, given that high touristic activity that the region has experienced in recent years is causing a low water quality combined with the increasing eutrophication due to the nutrients drained through the basins from the surrounding agricultural fields. These factors may probably be of high impact for the presence of the three other species of *Pisidium*; this impact may be related to changed ecological factors which require more intense studies to test this hypothesis.

In the present work, we present a first conclusive overview of the diversity and distribution of the species of Sphaeriidae in Morocco. The country very likely contains a western Palearctic relict fauna of sphaeriids, which, due to their small size, seem to be easily transported over long distances. Many European species have been found in North Africa and these findings are being tested in a phylogeographic context elsewhere. The faunistic and taxonomic data on Sphaeriidae and, particularly, on the genus *Pisidium* are scarce in Africa and even more so in North Africa. This is probably due to their small size, hidden mode of life and lack of clear diagnostic characters; therefore, listing the biodiversity of such a cryptic group may be impaired. Given this situation, more extensive future work is needed to study the ecology, life cycle patterns and the phylogeny of the species of Sphaeriidae.

## Supplementary Material

XML Treatment for Pisidium casertanum

XML Treatment for Pisidium subtruncatum

XML Treatment for Pisidium personatum

XML Treatment for Pisidium amnicum

XML Treatment for Pisidium sp.

XML Treatment for Musculium lacustre

1DBF41EB-D838-5765-862F-97AA0D6F22AC 10.3897/BDJ.9.e73346.suppl1 Supplementary material 1 Coordinates of occurrence sites of Sphaeriidae in Morocco with cross-marked presence of species per basin. Data type Coordinates Brief description Coordinates of occurrence sites of Sphaeriidae in Morocco with cross-marked presence of species per basin. DRA: Drâa-Ziz-Rhéris Basin, OER: Oum Er Rabia Basin, SEB: Sebou Basin, TEN: Tensift Basin, SM: Souss-Massa Basin, ML: Moulouya Basin, LK: Loukkos Basin, Pca: *P.casertanum*, Ppe: *P.* (cf.) *personatum*, Psu: *P.subtruncatum*, Pam: *P.amnicum*, Mla: *M.lacustre*. The cross is marked in bold at locations where *P.* (cf.) *personatum* occurred. File: oo_593768.docx https://binary.pensoft.net/file/593768 Hanane Rassam

## Figures and Tables

**Figure 9a.**
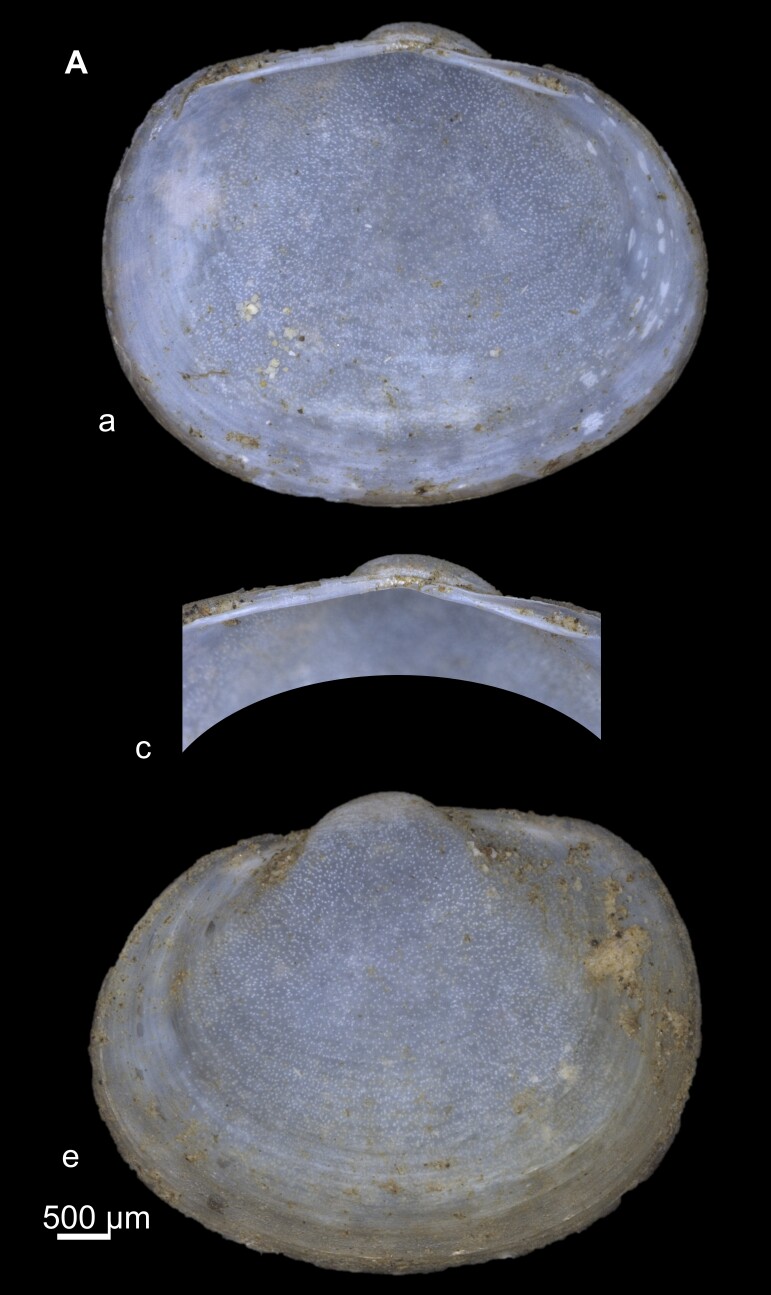


**Figure 9b.**
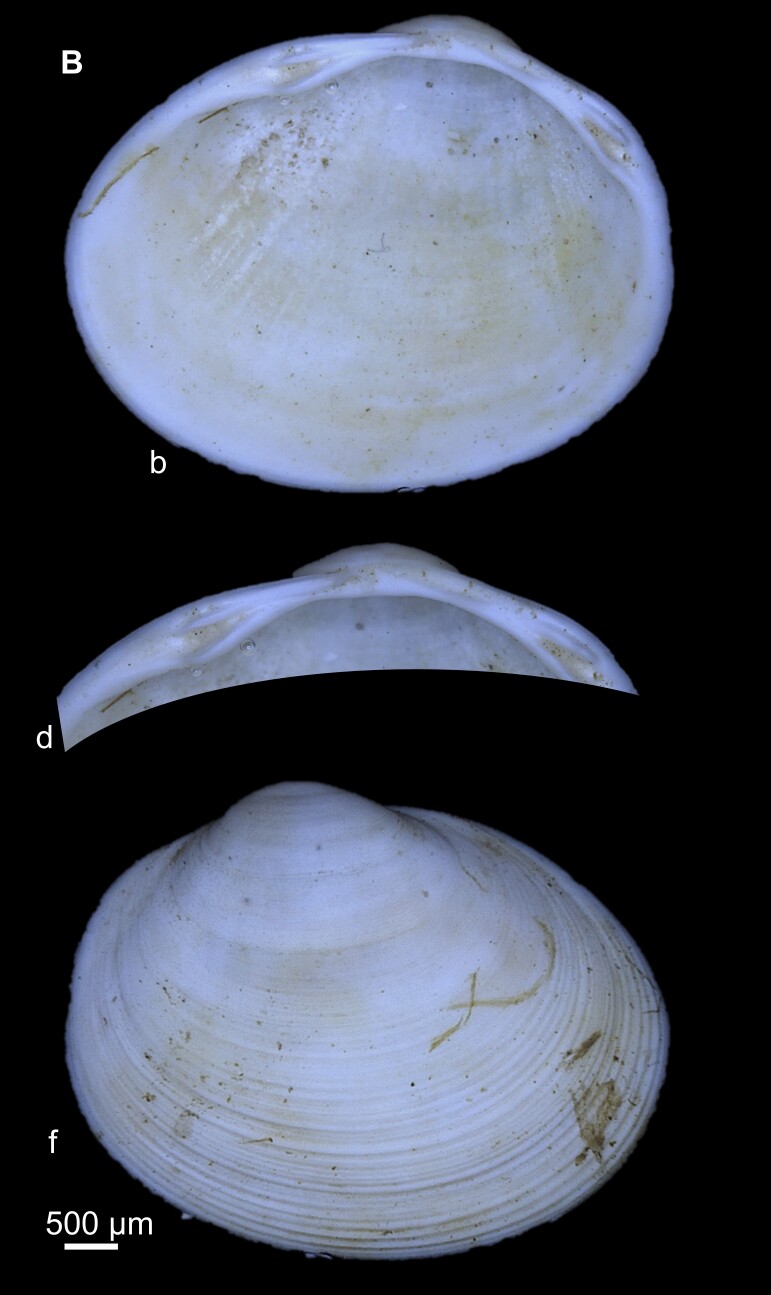


**Table 1.** Results of the regional conservation status assessment for three species of Sphaeriidae in Morocco. EOO: Extent of Occurrence, AOO: Area of Occupancy; no. unique occ.: number of unique occurrences; no. subPop.: number of subpopulations; no. loc: number of locations; LC: Least Concern; VU: Vulnerable; NA: Not applicable.

	*P.casertanum*	*P.* (cf.) *personatum*	*P.subtruncatum*	*P.amnicum*	*M.lacustre*
EOO (km^2^)	50,915	45,741	15,219	NA	NA
AOO (km^2^)	104	52	40	4	4
no. unique occ.	27	13	11	1	1
no. subPop.	16	13	8	1	1
no. loc	20	13	8	1	1
category criteria B	LC	LC	VU	CR	CR
category code	LC B1a+B2a	LC B1a+B2a	VU B1a+B2a	CR B2a	CR B2a
