# Supplementary material for: The fingernail clams (Bivalvia: Veneroida: Sphaeriidae) of Morocco: Diversity, distribution and conservation status
Source: Biodivers Data J. 2021 Dec 2;9:e73346. doi: 10.3897/BDJ.9.e73346 (PMC8671715; doi:10.3897/BDJ.9.e73346)
Supplement: Supplementary material 1 — Coordinates of occurrence sites of Sphaeriidae in Morocco with cross-marked presence of species per basin. [file bdj-09-e73346-s001.docx]

Coordinates of occurrence sites of Sphaeriidae in Morocco with cross-marked presence of species per basin. DRA: Drâa-Ziz-Rhéris basin, OER: Oum Er Rabia basin, SEB: Sebou basin, TEN: Tensift basin, SM: Souss-Massa basin, ML: Moulouya basin, LK: Loukkos basin, Pca: *P. casertanum,* Ppe*: P.* (cf.) *personatum,* Psu*: P. subtruncatum,* Pam*: P. amnicum,* Mla*: M. lacustre.* The cross is marked in bold at locations where *P.* (cf.) *personatum* occurred.

| Site | Longitude | Latitude | Basin | Pca | Ppe | Psu | Pam | Mla |
| --- | --- | --- | --- | --- | --- | --- | --- | --- |
| Télouet | 31°16’54’’N | 7°08’57.6"W | DRA | X |  |  |  |  |
| Zaouit Cheikh | 32°38'39.6''N | 5°54'48.8''W | OER | X |  | X |  |  |
| Sources O. Rbiaa | 33°03'11.41"N | 5°24'50.23"W | OER | X |  |  |  |  |
| Ouiouane | 33°07’48’'N | 5°21’10.2’’W | OER | X | X |  |  |  |
| Aguelmame Sidi Ali | 33°04’25.92"N | 5°00’11.28’'W | SEB | X |  |  |  |  |
| O. Guigou | 32°53'20.58"N | 5° 2'58.56"W | SEB | X |  | X |  |  |
| O. Mikkès | 33°33’44.4’'N | 5°07'25.8’’W | SEB | X |  |  |  |  |
| Hachlaf (outlet) | 33°34'50.1"N | 4°58'44.1"W | SEB |  |  | X |  | X |
| Hachlaf (dam) | 33°34'50.1"N | 4°58'44.1"W | SEB | X |  |  | X |  |
| Dayet Ifrah | 33°33'31.00"N | 4°55'47"W | SEB | X |  |  |  |  |
| Imlil | 31°03’54.6’'N | 7°56’14.4’'W | TEN | X |  |  |  |  |
| Tiferguine | 31°11'48.6"N | 7°50'03"W | TEN | X |  | X |  |  |
| Amghass1 | 33°22'52.32"N | 5°26'30.61"W | SEB | X | **X** |  |  |  |
| Aguelmame Azegza | 32°58'25.15"N | 5°26'42.24"W | OER | X |  |  |  |  |
| Oued Boulmane | 31°11'46.00"N | 6°10'7"W | DRA | X |  |  |  |  |
| Oued Mgoune | 31°22'25.00"N | 5°59'29"W | DRA | X |  |  |  |  |
| Zegmouzen | 30°31'60.00"N | 7°55'57"W | SM | X |  |  |  |  |
| Grotte Ifri N'Touya (Ait M'hamed) | 31°52'46.45"N | 6°27'01.87"W | OER |  |  | X |  |  |
| Grotte Ifri N'Touya | 31°52'46.45"N | 6°27'01.87"W | OER |  | X | X |  |  |
| Ouzioua | 30°44'11.00"N | 7°55'57"W | SM | X |  |  |  |  |
| Région Tighdouine | 31°24'28.12"N | 7°29'23.99"W | TEN | X |  |  |  |  |
| Source Tizirt | 31°17'38.11"N | 7°29'33.38"W | TEN | X |  |  |  |  |
| Oued Chbouka | 32°52'34.43"N | 5°22'10.21"W | OER |  |  | X |  |  |
| Source Chbouka | 32°52'48,34"N | 5°22'04,73"W | OER |  |  | X |  |  |
| Lac Miaami | 32°54'13.17"N | 5°22'45.30"W | OER |  |  | X |  |  |
| Source, route Bouleman st.3 | 33°29’58.25’’N | 5°04’42.06’’W | SEB | X |  | X |  |  |
| Tizguit (st.4) | 33°30’42.25’’N | 5°05’16.72’’W | SEB | X |  |  |  |  |
| Tizguit (st.5) | 33°33’25.8”N | 5°06’15.23’’W | SEB |  | X | X |  |  |
| Tizguit, Termila | 33°32’44.86”N | 5°06’20.17”W | SEB | X |  |  |  |  |
| Mikkès, route Zaouit Ifrane | 33°32’31.08”N | 5°06’54.73”W | SEB | X |  | X |  |  |
| Ain Vitel, en aval de la source | 33°32'50.27"N | 5°06'44.99"W | SEB | X |  |  |  |  |
| Ain Aghbal (Azrou) | 33°26'23.73''N | 5°14'47.65"W | SEB | X |  |  |  |  |
| Oued Tassaout | 31°24'57.91"N | 6°47'20.73"W | OER |  | **X** |  |  |  |
| Lac Isli | 32°12'49.84"N | 5°32'57.13"W | OER | X |  |  |  |  |
| Dayet Iffer | 33°36'23.04"N | 4°54'28.33"W | SEB | X |  |  |  |  |
| Source Ouayfirte | 31°26'17.3"N | 7°32'6.5"W | TEN |  | X |  |  |  |
| Ait Bououli, rte Ait Bouguemez | 31°36'45.47"N | 6°35'13.90"W | OER |  | X |  |  |  |
| Ait Bouguemez | 31°38'25,49"N | 6°28'40.75"W | OER | X | X |  |  |  |
| Rte entre Ighil et Tanmrt | 31°41'29.85"N | 6°32'14.71"W | OER | X |  |  |  |  |
| Source Mlaeb | 34° 3'40.92"N | 4°9'51.64"W | SEB | X |  |  |  |  |
| Source Kawan | 34°6'37.49"N | 4°19'13.61"W | SEB | X |  |  |  |  |
| Tazlida | 31°25'35"N | 7°24'39"W | TEN | X |  |  |  |  |
| Tizguit | 33°35’8.376’’N | 5°09’2.376’’W | SEB | X |  |  |  |  |
| Route Chhida | 33°24’19.44’’N | 5°23’13.2’’W | SEB | X |  |  |  |  |
| Rte Sidi Addi | 33°23'58.71''N | 5°19'33.05''W | SEB |  | **X** |  |  |  |
| Oued Tigrigra | 33°25’31.51’’N | 5°16’23.88’’W | SEB | X |  |  |  |  |
| Ain Sultan | 33°43’15.6’’N | 5°00’17.71’’W | SEB |  | **X** |  |  |  |
| Ain Fendel | 34°05’44.41’’N | 4°26’24.36’’W | SEB | X |  | X |  |  |
| Source Tamda | 31°18'50.74"N | 7°3'53.89"W | DRA |  | **X** |  |  |  |
| Rte Aghbalou N'Serdane | 32°41'00.1"N | 5°32'55.3"W | SEB | X |  |  |  |  |
| L'ksabi | 32°50'06.88"N | 4°24'37.26"W | MOL | X |  |  |  |  |
| Oued Majjo | 35°06'20.15"N | 5°11'32.19"W | LK |  | X |  |  |  |
| Grotte Izoura | 34°05'41.00"N | 4°05'55.72"W | SEB |  | X |  |  |  |
| Séguia région Ain Taoujdat | 33°53'35.29"N | 5°14'30.06"W | SEB | X |  |  |  |  |
| Séguia région Chefchaoun | 35°6'10.164"N | 5°10'12.954"W | LK | X |  |  |  |  |
